# Multifunctional application of PVA-aided Zn–Fe–Mn coupled oxide nanocomposite

**DOI:** 10.1186/s11671-020-03464-0

**Published:** 2021-01-02

**Authors:** Buzuayehu Abebe, H. C. Ananda Murthy, Enyew Amare Zereffa

**Affiliations:** Department of Applied Chemistry, School of Applied Natural Science, Adama Science and Technology University, P O Box 1888, Adama, Ethiopia

**Keywords:** Porous ternary nanocomposite, Adsorption, Photocatalysis, Antibacterial activity, Sensor, Mechanisms

## Abstract

Zinc oxide (ZnO) is a fascinating semiconductor material with many applications such as adsorption, photocatalysis, sensor, and antibacterial activities. By using a poly (vinyl alcohol) (PVA) polymer as a capping agent and metal oxides (iron and manganese) as a couple, the porous PVA-aided Zn/Fe/Mn ternary oxide nanocomposite material (PTMO-NCM) was synthesized. The thermal, optical, crystallinity, chemical bonding, porosity, morphological, charge transfer properties of the synthesized materials were confirmed by DTG/DSC, UV–Vis-DRS, XRD, FT-IR, BET, SEM-EDAX/TEM-HRTEM-SAED, and CV/EIS/amperometric analytical techniques, respectively. The PTMO-NCM showed an enhanced surface area and charge transfer capability, compared to ZnO. Using the XRD pattern and TEM image analysis, the crystalline size of the materials was confirmed to be in the nanometer range. The porosity and superior charge transfer capabilities of the PTMO-NCM were confirmed from the BET, HRTEM (IFFT)/SAED, and CV/EIS analysis. The adsorption kinetics (adsorption reaction/adsorption diffusion) and adsorption isotherm test confirmed the presence of a chemisorption type of adsorbate/methylene blue dye-adsorbent/PTMO-NCM interaction. The photocatalytic performance was tested on the Congo red and Acid Orange-8 dyes. The superior ascorbic acid sensing capability of the material was understood from CV and amperometric analysis. The noble antibacterial activities of the material were also confirmed on both gram-negative and gram-positive bacteria.
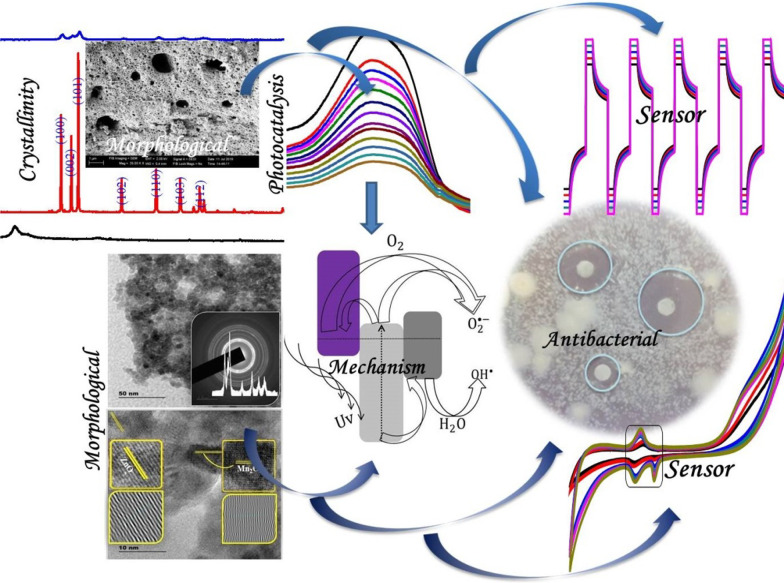

## Introduction

Zinc oxide nanoparticles (NPs) are commonly used in several fields such as adsorption [1], photocatalysis [2, 3], food preservation [4], and pollutant sensor [5]. Compared to TiO_2_, the production cost of ZnO is approximately 75% lower and has higher absorption efficacy across a large fraction of the solar spectrum [6, 7]. The application of single metal oxide as a photocatalyst is restricted on the charger transfer property due to the photogenerated electron/hole recombination. This recombination, particularly in the nanosized range, leads to the diminution of their quantum efficiency and also may lead to the dissipation of radiant energy by initiating highly desirable reactions [8, 9]. Among several efforts applied to reduce the electron–hole recombination problem such as doping, heterojunction, dye sensitization, noble and non-noble metal deposition, forming heterostructure materials was found to be one of the noble preferences [10–12]. Coupling of ZnO with other metal oxides was reported for remediation of the mentioned recombination problem [8, 13–16]. Due to their stability and unique properties, the hematite (α-Fe_2_O_3_) [8, 14] and Mn_2_O_3_ [13] are suggested to act as a decent couple with ZnO.

Besides, PVA polymer as a stabilizing agent also has great use in diminishing the electron–hole recombination problems [17]. As reported [18, 19], 500 °C is the optimum temperature to remove unwanted impurities including the PVA polymer after acting as a capping agent. Modifying the synthesized materials to have a mesoporous property that allows a rapid charge transfer process has been also reported [20, 21]. Using only environmentally benign water as a solvent and developing an efficient synthesis procedure, the toxicity, cancer-causing ability, and mutagenic properties of organic solvents can also be removed.

A small variation in the standard level of ascorbic acid creates a lot of diseases in human beings [16]. As reported [22], ascorbic acid has a major role in the physiological normal functioning of organisms and also used as a treatment for a different illness. Therefore, it is significant to develop novel methods used for measuring the level of ascorbic acid. Nowadays, metal oxide nanomaterials have been largely employing as sensor applications [23]. Among several techniques that have been made to improve the sensing properties of ZnO, forming a composite with other metal oxides and modifying the synthesized materials to have a mesoporous property that allows a rapid charge transfer process have been reported [20, 21]. Furthermore, hospital-acquired infections caused by microorganisms are becoming worldwide problems [24]. ZnO is also listed as an antimicrobial agent and safe material for food preservation of foodborne diseases by the US FDA (21CFR182.8991) [4, 25].

Considering all the mentioned aspects of aggregation/agglomeration, surface area-to-volume ratio, and toxicity of organic solvents, this work synthesizes PVA-assisted PTMO-NCM using a simple sol–gel followed by accidental self-propagation techniques. The as-synthesized material was characterized by DTG/DSC, XRD, BET, SEM–EDX/TEM/HRTEM/SAED, and CV/EIS/amperometric analytical techniques. A pronounced surface area and charge transfer capability improvement have been achieved for PTMO-NCM, compared to ZnO. The applicability of the synthesized coupled PTMO-NCM was tested on adsorption and degradation of organic dyes, antibacterial activity, and an ascorbic acid sensor.

## Materials and methods

The instrumental details and the reagents used were present as supplementary material (S). The detailed ZnO and PTMO-NCM synthesis procedures were also present in the author’s earlier works [1, 26–28]. Roughly, the PVA polymer was dissolved in distilled water with continuous stirring on a magnetic stirrer at ~ 115 °C for about 15 min. Then, the salt precursors, Zn(NO_3_)_2_.6H_2_O, Fe(NO_3_)_3_.9H_2_O, and MnSO_4_.H_2_O were mixed with previously dissolved and cooled PVA polymer solution with continuous stirring. After two days of aging followed by drying in an oven at about 110 °C, the product was gently crushed to reduce the highly amorphous self-propagated material. Finally, it was calcined at the DTG-optimized calcination temperature of 500 °C for 3 h. The calcination process at the optimized temperature helps for removing unwanted impurities as well as the PVA polymer. The synthesized PTMO-NCM was used for continuous sample characterization and application tests. The photocatalytic experiment was performed using a 176.6-cm^2^ circular glass reactor under a 125-W mercury vapor lamp. The 20 ppm of 250 mL Congo red (CR) and Acid Orange-8 (AO8) dyes and 0.06 g of PTMO-NCM photocatalyst were used during the experiment. The adsorption test was conducted using the experimentally optimized [1] adsorption parameters, 10–150-min adsorbate–adsorbent contact time, and 1–35 mg L^−1^ concentrations with a constant 140 rpm shaking speed. The antibacterial activity test had conducted using three different concentrations (75, 100, and 125 μg mL^−1^) of ZnO and PTMO-NCM. The experiment was accompanied by a disk diffusion method using a 0.5 McFarland standard.

## Results and discussion

### Characterization results

**Fig. 1:**
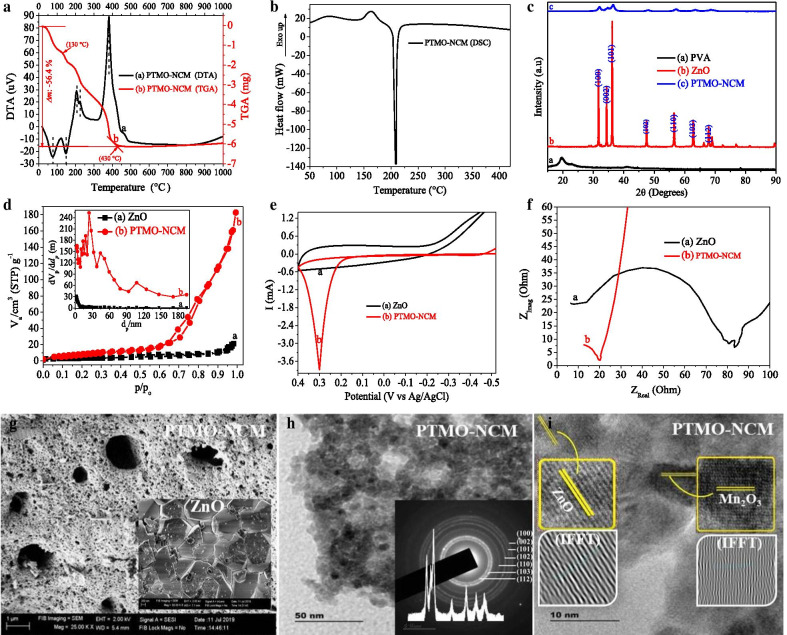
**a** DTG. **b** DSC. **c** XRD. **d** BET. **e** CV. **f** EIS plots. **g** SEM. **h** TEM. **i** HRTEM images of single ZnO and ternary nanocomposite materials

The optimum calcination temperature was determined to be 500 °C using DTG stability analysis at a 50 °C min^−1^ flow rate of nitrogen gas. About 56% of the sample decomposition took place and left with ~ 42% of pure PTMO-NCM (Fig. 1a). From the DSC plot (see Fig. 1b), the two exothermic peaks are supposed to be due to the evaporation of adsorbed volatile components at 80 °C and conformational changes at 144 °C. The third endothermic peak that appeared at about 210 °C is probably due to the phase transformation of other forms of iron or/and manganese oxides to the stable Fe_2_O_3_ and Mn_2_O_3_ phase. Compared to ZnO, the high reflectance drop in the visible region for PTMO-NCM was observed from UV–Vis-DRS spectroscopic analysis (Additional file 1: Fig. S1a). This optical analysis supports the peak intensity reduction of the XRD pattern and the porosity interpretation of the SEM image. The Kubelka–Munk plots [29, 30] showed the nonexistence of bandgap change between ZnO and PTMO-NCM (Additional file 1: Fig. S1b).

The noticeable approximate average crystalline size reduction (6×) was obtained for PTMO-NCM, compared to ZnO (Fig. 1c). The XRD pattern peaks of both ZnO and PTMO-NCM are consistent with the hexagonal ZnO phase (ICSD: 00-036-1451, P63mc (#186-1) space group). This is probably due to the smaller percentages of iron (5%) and manganese (5%) oxides. The absence of PTMO-NCM peaks shift relative to ZnO also shows the non-appearance of structural distortion on ZnO lattice. This may indicate the presence of only a local heterojunction between the ternary metal oxides [8, 31, 32]. The XRD data and the respective size of the particles were calculated using Debye–Scherrer’s formula (*D* = *Kλ*/(*β*cos(*θ*)), where λ is the wavelength of X-ray radiation (for Cu 0.15418 nm), *K* is constant close to unity, *β* is the full width at half maximum (FWHM) in 2*θ* scales and *θ* is the angle of the considered Bragg reflection [33, 34].

Compared to ZnO, the great surface area enhancement for PTMO-NCM (15×) and the porous nature of PTMO-NCM was approved from the BET and SEM image analysis, respectively (see Fig. 1d, g, (the inset image in Fig. 1g is for ZnO)). As per IUPAC classifications, among six types of adsorption isotherms (I–VI) and four types of the hysteresis loops, the BET plots of ZnO and PTMO-NCM look a typical IV isotherm and an H3 hysteresis loop. The approximate average BJH pore size distribution for ZnO and PTMO-NCM was determined to be 9 and 26, respectively, which is consistent with the mesoporous range of IUPAC classification [35]. The greater current rise in CV analysis [36] (Fig. 1e) and the smaller semicircle diameter of the Nyquist plot in EIS techniques [37] (Fig. 1f) confirm the enhanced charge transfer capabilities of PTMO-NCM over ZnO. The nanometer range crystalline size of the PTMO-NCM was further confirmed from the TEM image (Fig. 1h). The predictable composition and actuality of the PTMO-NCM were characterized by EDX (see Additional file 1: Fig. S2) and HRTEM analysis (Fig. 1i and its insets), respectively. The d-spacing values (0.2864, 0.2543, 0.1969, 0.1663, 0.1520, 0.1419, and 0.1104) that was determined from SAED rings (Fig. 1h inset) are also matching with XRD pattern result. The stacking faults on the HRTEM (IFFT) image and the nonexistence of the diffraction spots in the SAED ring that confirms the crystallinity of the materials [38] further confirms the porous nature of the PTMO-NCM.

### Methylene blue dye adsorption

**Fig. 2:**
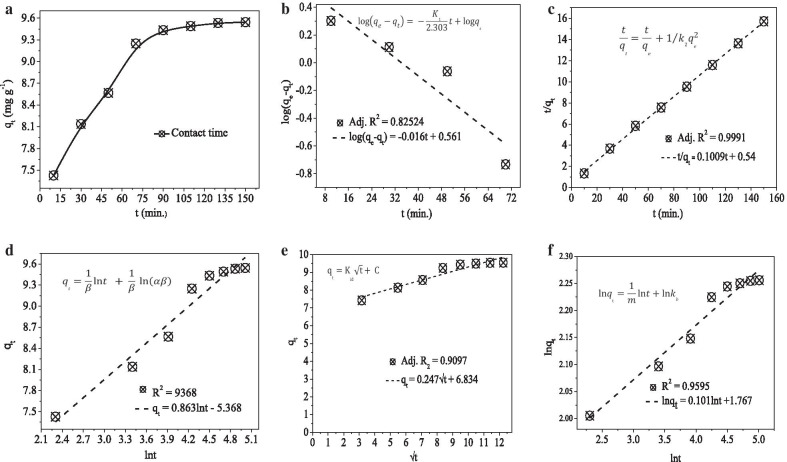
**a** Adsorption kinetics plot. **b** Pseudo-first-order. **c** Pseudo-second-order. **d** Elovich. **e** Intraparticle diffusion. **f** Bangham kinetics models

The optimized 0.02 g dosage, pH of 8, and a constant 140 rpm shaking speed were used for the adsorption-reaction and adsorption-diffusion kinetics studies [1]. The coefficient of determination (*R*^2^) value and equations used to calculate the adsorption kinetics models parameter was given in the respective plots as inset (Fig. 2). Among the pseudo-first-order (PFO) (Fig. 2b), pseudo-second-order (PSO) (Fig. 2c), and Elovich (Fig. 2d) adsorption-reaction models, the PSO model that confirms the chemisorption types of adsorption fits well. Also, the theoretical (9.43 mg g^−1^) and experimental (9.91 mg g^−1^) values of the PSO model have a close relation unlike that of the PFO that has the experimental values of (3.64 mg g^−1^). The intraparticle diffusion (IPD) model seems fitting well (Fig. 2e); however, to say the reaction is under the control of adsorption-diffusion, its linear plot should pass through the origin. The IPD plot for this work is not passing through the origin. From this, it is possible to conclude that the reaction is dominantly under the control of adsorption-reaction. However, the well-fitting of the Bangham model (Fig. 2f) is indicating the presence of pore diffusion in the adsorption process [39]. The presence of this pore diffusion is also consistent with the BET and SEM interpretations.

**Fig. 3:**
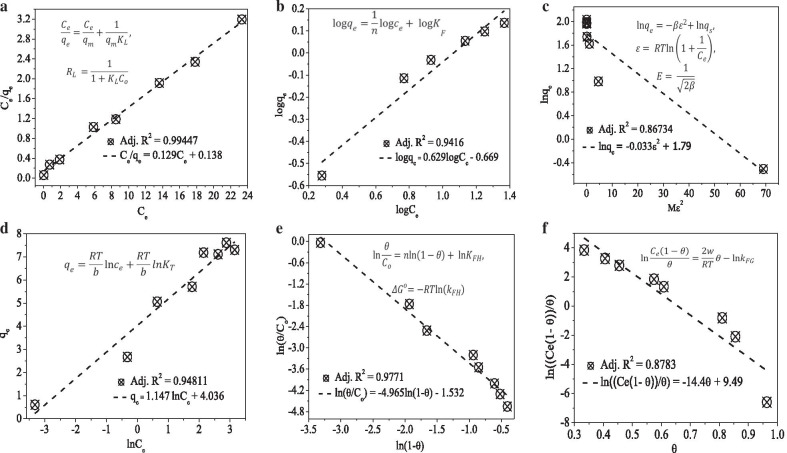
Adsorption isotherm plots of **a** Langmuir. **b** Freundlich. **c** Dubinin–Radushkevich. **d** Temkin. **e** Flory–Huggins. **f** Fowler–Guggenheim models

The *R*^2^ value and equations used to calculate the adsorption isotherm models parameter were also given in the respective plots as inset (Fig. 3). Depending on the *R*^2^ values of the adsorption isotherm models (Langmuir (Fig. 2a), Freundlich (Fig. 2b), Dubinin–Radushkevich (D–RK) (Fig. 2c), Temkin (Fig. 2d), Flory–Huggins (FH) (Fig. 2e), and Fowler–Guggenheim (FG) (Fig. 2f)), the Langmuir and FH models are showing relatively better fitting. From the Langmuir model, lying the separation factor *R*_L_ value between 0 and 1 (0.05) indicates the favorability of the adsorption process. The favorability of the adsorption process was also further confirmed from the n (1.59) value of the Freundlich model. The well-fitting of the Langmuir model indicates the presence of a monolayer methylene blue dye coverage, which is consistent with the PSO kinetics model interpretation. The maximum adsorption capacity of the adsorbent that was determined from the Langmuir isotherm model is 7.75 mg g^−1^. The indication of the characteristic surface coverage and spontaneity of the reaction (− 3.8 kJ mol^−1^) were also deduced from the FH model equation.

### Congo red and Acid Orange-8 dye degradation and mechanism

**Fig. 4:**
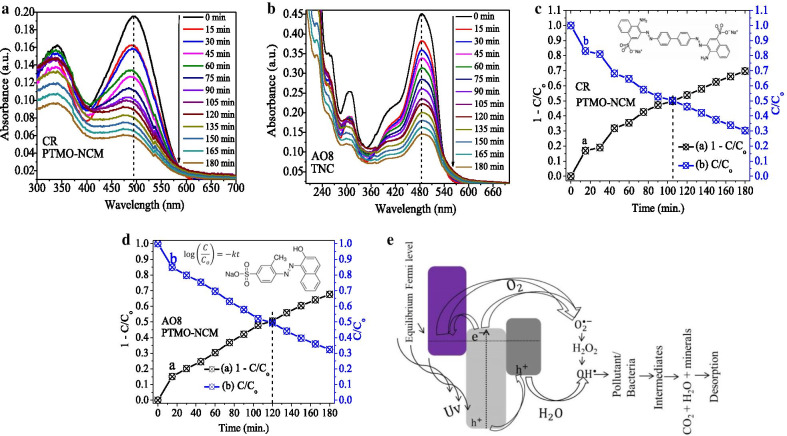
Photocatalytic activities of the PTMO-NCM: **a**, **b** absorbance vs. wavelength plots. **c**, **d** 1 − *C*/*C*_o_ versus *t* and *C*/*C*_o_ versus *t* plots of CR and AO8, respectively. **e** Proposed mechanism

The photodegradation capabilities of PTMO-NCM were studied on the decolorization of CR and AO8 dyes at a maximum absorption wavelength of 494 and 484 nm (Fig. 4a, b), respectively. In the first 15 min, approximately 17% of CR dye and 15% of AO8 dye degradation took place. At 180 min, the maximum degradation of 70% for CR dye and 68% for AO8 dye was taking place. The obtained equilibrium constant *k* values for CR and AO8 dyes were 0.007141 and 0.005627 min^−1^, respectively. From the contact point of 1 − *C*/*C*_o_ versus *t* and *C*/*C*_o_ versus *t* plots (see Fig. 4c, d), the obtained degradation half-life value was approximately 105 min for CR and 119 min for AO8. See the PFO kinetic equation used to study reaction dynamics in Fig. 4d inset.

The band edge position of metal oxides is highly dependent on the surface charge. For effective photocatalytic reaction, the bottom of the CB needs to be more negative than the redox potential of H^+^/H_2_ and the top of the VB needs to be more positive than the redox potential of O_2_/H_2_O [40, 41]. As reported [13], the CB of Mn_2_O_3_ and ZnO is close to each other. Besides, for confirming the presence of an appropriate heterojunction and reality of the proper charge transfer synergy, analysis using electrochemical techniques such as CV and EIS is significant [42]. As seen in the CV (Fig. 1e) and EIS (Fig. 1f) analysis, the PTMO-NCM is showing the presence of a suitable heterojunction. Therefore, the possible photocatalytic mechanism was proposed as seen in Fig. 4e. During heterojunction, until the Fermi level equalizes, the energy band of metal oxides starts to move up and down by transferring electrons [8, 43] and lead to the creation of a depletion layer in the interface [44]. The Fermi level of p-type Mn_2_O_3_ exists near the VB. During UV irradiation, the photogenerated electrons have the probability of either localizing on the ZnO CB or diffusing to the VB of the Mn_2_O_3_, and the holes move to the VB of Fe_2_O_3_. Therefore, the recombination of the electrons and holes diminished and resulted in enhanced photocatalytic activity [8].

**Fig. 5:**
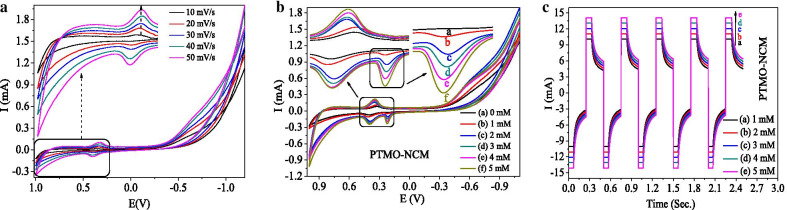
**a** CV plots at different scan rates. **b** CV ascorbic acid sensing curve at different concentrations. **c** Amperometric ascorbic acid sensing plot at different concentrations

From the CV graph of PTMO-NCM (Fig. 5a), the reduction-reaction peaks were observed. As reported [45], this fast and reversible redox reaction is indicated to be due to the porous nature of the materials. This is also consistent with the BET and SEM characterization results. The obtained approximate peak potential difference (Δ*E*_a,c_) between *E*_pa_ (+ 0.401 V) and *E*_pc_ (+ 0.323 V) peak is 0.078 V. This smaller Δ*E*_a,c_ value shows the capability of the PTMO-NCM material to be more reversible. With an increase in the scan rate, the redox peaks positively shifted towards anodic and cathodic potentials. As seen in Fig. 5b CV plot and Fig. 5c amperometry plot, the novelty of the PTMO-NCM as an ascorbic acid sensor was also confirmed, as the concentration of ascorbic acid increase results in increasing the current rise. The sensing nobility of the material was also confirmed from the amperometry analysis as the sensing cycle was completed within a few seconds. The cycles were repeated to evaluate the stability of the electrode for 1 h. The obtained result confirms the stability and reproducibility of the PTMO-NCM electrode.

**Fig. 6:**
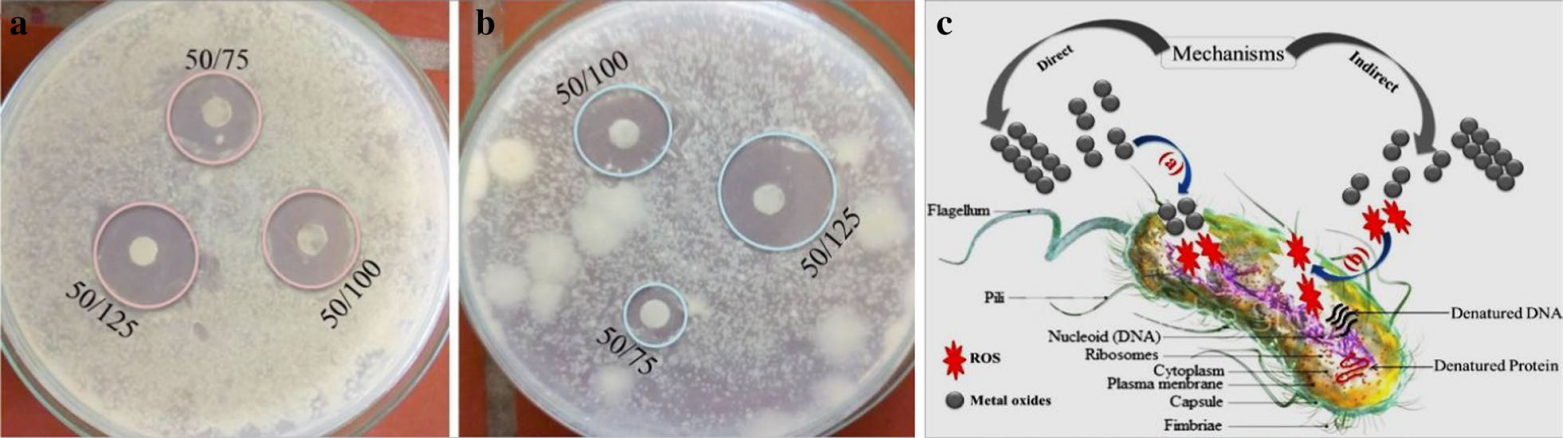
The antibacterial activity of PTMO-NCM towards **a** *E. coli.* **b** *S. aureus. c* antibacterial mechanism (50/75: 50 is the percentage of PTMO-NCM during synthesis, 75 is the amount used in μg/mL during antibacterial activity)

The antibacterial activity of metal oxides is highly dependent on the particle size [46] and ROS [47] generation capacities of the materials. By taking different precursor percentages and PVA polymer amount [26], the optimum antibacterial activities of PTMO-NCM towards *E. coli* and *S*. *aureus* (Fig. 6a, b, respectively) were determined to be 50% ZnO, 25% Fe_2_O_3_, and 25% Mn_2_O_3_. The enhanced antibacterial activities for PTMO-NCM were achieved compared to both single ZnO- and binary ZnO-based materials [27]. The antimicrobial activity mechanism of NPs may follow three mechanisms [48], including the release of antimicrobial ions [25, 49], the interaction of NPs with microorganisms [50], and the formation of ROS by the effect of light radiation [51]. As confirmed from the XRD pattern and UV–Vis-DRS spectra, the structural distortion and band position shift had not observed. The absence of this distortion and shift is due to the non-intercalation of Fe^3+^/Mn^3+^ ions. This indicates the antimicrobial activity due to ions may not be the proper mechanism. Therefore, the direct and indirect ways of ROS generation [52] were proposed as an antibacterial activities mechanism, as seen in Fig. 6c.

## Conclusions

The PTMO-NCM that has high porosity, enhanced surface area, and superior charge transfer capability was synthesized using the sol–gel followed by self-propagation techniques. Using the XRD pattern and TEM image analysis, the approximate average crystalline size of PTMO-NCM was determined to be in the range of 10–60 nm. The crystalline size of PTMO-NCM is six times smaller than bare ZnO. Compared to ZnO, fifteen times surface area enhancement for PTMO-NCM was confirmed from BET analysis. The less crystalline nature of the PTMO-NCM further confirmed from the stacking faults present on the HRTEM (IFFT) image and the absence of diffraction spots on the SAED ring. The nine times smaller semicircular diameter on the EIS and an enhanced current rise on CV indicate the presence of novel charge transfer properties for PTMO-NCM, compared to ZnO. From the adsorption kinetics and adsorption isotherms study, the adsorbate–adsorbent interaction was examined to be a chemisorption type. From the Langmuir model, the maximum adsorption capacity was determined to be 7.75 mg g^−1^. The photocatalytic equilibrium constants were found to be 0.007141 min^−1^ and 0.005627 min^−1^ for CR and AO8 dyes, respectively. The superior sensing capability and noble antibacterial activities of PTMO-NCM were also verified.

## Supplementary Information

**Additional file 1: 1.** Reagents. **2.** Materials and Instrumental details. **Figure S1.** a DRS-UV-vis. b direct Kubelka–Munk (Inset b: the respective indirect plots). c FT-IR spectra. **Figure S2.** EDX spectra (inset elemental percentage compositions).

## Data Availability

The datasets used and/or analyzed during the current study are available from the corresponding author on reasonable request.

## Abbreviations

PTMO-NCM: Porous ternary metal oxide nanocomposite material
UV*–*Vis-DRS: UV*–*Vis-diffuse reflectance spectroscopy
FT-IR: Fourier transform infrared spectroscopy
XRD: X-ray powder diffraction
SEM: Scanning electron microscopy
EDX: Energy-dispersive X-ray spectroscopy
TEM: Transmission electron microscopy
HRTEM: High-resolution transmission electron microscopy
SAED: Selected area electron diffraction
BET: Brunauer–Emmett–Teller
CV: Cyclic voltammetry
EIS: Electrical impedance spectroscopy
FH: Flory–Huggins
FG: Fowler–Guggenheim
PFO: Pseudo-first-order
PSO: Pseudo-second-order
IPD: Intraparticle diffusion
CR: Congo red
AO8: Acid Orange-8
IFFT: Inverse fast Fourier transmission
ROS: Reactive oxygen species
*S. aureus*: *Staphylococcus aureus*
*E. coli*: *Escherichia coli*
