# Supplementary material for: Multifunctional application of PVA-aided Zn–Fe–Mn coupled oxide nanocomposite
Source: Nanoscale Res Lett. 2021 Jan 2;16:1. doi: 10.1186/s11671-020-03464-0 (PMC7778673; doi:10.1186/s11671-020-03464-0)
Supplement: Supplementary file 1 — Additional file 1: 1. Reagents. 2. Materials and Instrumental details. Figure S1. a DRS-UV-vis. b direct Kubelka–Munk (Inset b: the respective indirect plots). c FT-IR spectra. Figure S2. EDX spectra (inset elemental percentage compositions). [file 11671_2020_3464_MOESM1_ESM.docx]

**Additional file 1**

**1. Reagents**

The common analytical grade reagents used are zinc nitrate hexahydrate (Zn(NO_3_)_2_.6H_2_O) (≥ 98%, Sigma-Aldrich); iron nitrate nonahydrate (Fe(NO_3_)_3_.9H_2_O) (99.95%, Sigma-Aldrich); manganese sulfate (MnSO_4_.H_2_O, 99%, Sigma-Aldrich); poly(vinyl alcohol) (PVA) (Thermo Fisher Scientific India Pvt. Ltd, 99% hydrolyzed); sodium hydroxide (NaOH) (98%, Oxford Lab Fine Chem LLP)_,_ Congo red (Sigma-Aldrich, pH of 8-9.5); acid orange-8 (AR8) (Sigma-Aldrich, pH of 11-13); and ascorbic acid (Sigma-Aldrich 99% purity).

**2. Materials and Instrumental details**

DTG (DTG-60H) and DSC (PerkinElmer, DSC 4000, USA), Ultraviolet-visible spectroscopy techniques (UV-Vis) (SM-1600), Fourier transform-infrared spectroscopy (FT-IR) (Spectrum 65 FT-IR (PerkinElmer)) using KBr pellets, scanning electron microscopy with energy-dispersive X-ray spectroscopy (SEM-EDX-EVO 18 model with low vacuum facility and ALTO 1000 cryo attachment), transmission electron microscope and high-resolution transmission electron microscope (JEOL TEM 2100 HRTEM), N_2_ adsorption-desorption (Quantachrome instrument), X-ray diffraction (XRD-Shimadzu X-Ray Diffractometer (PXRD-7000)). For electrochemical properties investigation: the CV, EIS, and amperometric (CHI604E potentiostat) studies were conducted using a tri-electrode system. The ZnO and optimized BMONCs and TMONCs were used as a working electrode, whereas the platinum wire as a counter electrode, and Ag/AgCl as a reference electrode with 6.0 M KOH electrolyte. The EIS studies were carried out in the frequency range of 1 Hz and 1 MHz, with an AC amplitude of 5 mV. The other common instruments used for this work include photoreactor (176.6 cm^2^ circular glass reactor under 125 W mercury vapor lamp); pH meter (MP 220); magnetic stirrer; shaker (incubating orbital shaker SO1 made in the UK) hot air oven (Contherm 260 M); and furnace.

**Figures**

**Fig. S1** **a** DRS-UV-vis. **b** direct Kubelka–Munk (Inset **b**: the respective indirect plots). **c** FT-IR spectra.


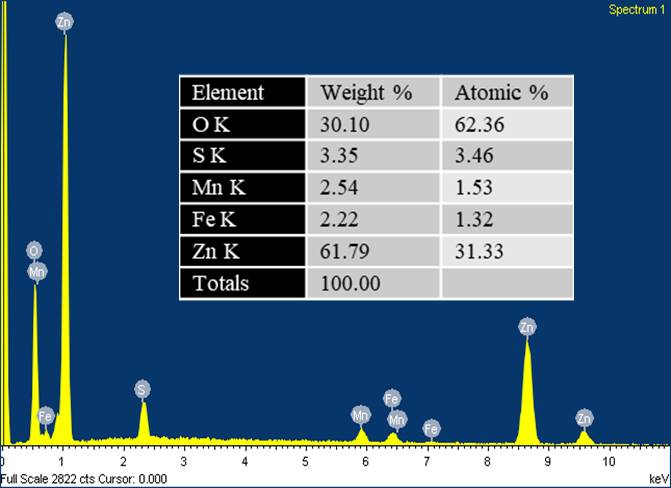


**Fig. S2**. EDX spectra (inset elemental percentage compositions).
